# Vibration evolution and fault risk assessment of deep-sea drilling fluid lift pumps

**DOI:** 10.1038/s41598-026-51472-4

**Published:** 2026-05-06

**Authors:** Rulei Qin, Xuelian You, Changping Li, Yanjiang Yu, Wenwei Xie

**Affiliations:** 1 School of Ocean Sciences, China University of Geosciences (Beijing), Beijing, 100000 China; 2 Institute of Exploration Techniques, Chinese Academy of Geological Sciences, Langfang, 065000 China; 3 Technology Innovation Center for Directional Drilling Engineering, Ministry of Natural Resources, Langfang, China; 4 School of Mechanical Engineering and Electronic Information, China University of Geosciences, Wuhan, 430074 China; 5 Guangzhou Marine Geological Survey, Guangzhou, 510000 China

**Keywords:** Drilling fluid lift pump, Fluid-structure Interaction, Dynamic analysis, Vibration characteristics, Fault identification, Energy science and technology, Engineering

## Abstract

The vibration stability of the drilling fluid lifting pump significantly impacts the safety and efficiency of deep-sea drilling. This study establishes a Fluid-Structure Interaction (FSI) dynamic analysis model of a six-stage centrifugal lift pump to analyze the excitation force characteristics and modal response under fluid action. A vibration test platform was constructed to conduct head-flow (H-Q) performance tests and investigate vibration characteristics across the full flow range (0–260 m³/h). A joint evaluation method based on the Root Mean Square (RMS) of vibration velocity and the Crest Factor (Cf) of acceleration is proposed. Combined with time-domain waveform characteristics, this method accurately identifies vibration risks and faults under off-design condition. The results indicate that the lift pump operates stably under rated conditions (1450 r/min, 126 m³/h). However, when the flow rate deviates from the rated value, the vibration exhibits strong nonlinear characteristics, and fault risk increases significantly. The optimal operating flow range for the pump under off-design conditions is determined to be 130–156 m³/h. This study provides a theoretical basis and engineering guidance for vibration optimization and fault prevention of multistage centrifugal pumps in deep-sea applications.

## Introduction

The ocean contains vast oil and gas resources, with proven potential reserves exceeding one-third of the global total, making it a crucial future domain for energy supply^1^. However, as oil and gas exploration extends into deep and ultra-deep waters, drilling operations encounter severe technical challenges^2,3^. Firstly, the complex deep-sea environment (e.g. high pressure, low temperature) imposes extremely high demands on drilling equipment, directly causing to a sharp increase in operational costs. Secondly, subsea formations often exhibit a narrow fracture pressure window and a narrow safe mud weight window, requiring more casing strings to maintain wellbore stability, which significantly increasing operational complexity^4–6^. Dual-gradient drilling technology can effectively widen the safe density window of drilling fluid by precisely controlling the pressure distribution within the wellbore, thereby significantly enhancing well control safety and protect hydrocarbon reservoirs. It has become a key development direction in deepwater drilling^7^. Among these technologies, the Riserless Mud Recovery (RMR) system is widely recognized as the core technical solution for achieving dual-gradient drilling^8,9^. Within the RMR system, the deep-sea drilling fluid lift pump serves as the core power component. Typically constructed by connecting multistage centrifugal pumps in series, it performs the critical task of lifting the drilling fluid with cuttings from the seabed to the drilling platform. Its operating performance directly determines the accuracy of dual-gradient pressure control and the reliability of the entire drilling system^10,11^. Moreover, maintaining a stable pressure–flow environment is essential for wellbore safety in weakly consolidated deep-sea formations, where pressure disturbance may increase the risk of wellbore leakage and integrity deterioration^12^. However, due to its complex structure, multistage centrifugal pumps are susceptible to a wide range of technical vibration-related faults in extreme deep-sea conditions. These faults include not only mechanical component issues, such as rotor unbalance, misalignment, and mechanical looseness but also complex phenomena induced by fluid vibrations, such as pressure pulsations, cavitation, and vortex shedding^13,14^. Tong^15^ pointed out that the coupled effect of multiple faults significantly accelerates performance degradation and vibration. Tan^16^ also found that multiple faults lead to a significant decrease in pump head and efficiency. If these vibration issues are not effectively controlled, it can not only cause performance degradation and efficiency loss of the pump unit but also lead to fatigue damage, component failure, or even catastrophic failure incidents, directly threatening the safety operation of the entire system and significantly shortening the equipment lifecycle. Therefore, an in-depth investigation of the vibration characteristics of deep-sea drilling fluid lift pumps, along with systematic research on risk assessment and fault identification under multiple operating conditions, holds important theoretical significance and engineering value for improving the reliability and safety of deep-sea drilling equipment and ensuring efficient operation.

Centrifugal pumps endure fluid excitation during operation, and scholars worldwide have conducted multiple studies on the unsteady flow within centrifugal pumps^17,18^. Many studies have shown that unsteady flow structures, such as separation, stall, wake development, and vortex evolution are major sources of pump vibration^19–24^. Second, modal and fluid-structure interaction analyses have confirmed that hydraulic loads can significantly affect natural frequencies, mode shapes, and resonance behavior, especially in multistage rotor systems ^25–30^. Research on the flow-induced vibration characteristics of centrifugal pumps primarily relies on high-precision numerical simulations and advanced experimental measurement techniques, widely employing Fluid-Structure Interaction (FSI) technology to analyze the complex interactions between the fluid and the structure within the pump^31–33^. Chen^34^ investigated the vibration characteristics caused by cavitation in a single-blade centrifugal pump through combined numerical simulation and experimental analysis. Bolat^35^ systematically evaluated the dynamic response and vibration characteristics of a multistage centrifugal pump by integrating Computational Fluid Dynamics (CFD), rotor dynamics analysis, and experimental validation. Li^36^ experimentally explored the cavitation vibration characteristics of a centrifugal pump at normal flow rate. Li^37^ combined Computational Fluid Dynamics (CFD) and cyclostationarity analysis to reveal the modulation mechanism of flow-induced effects in centrifugal pump vibration signals. Wang^38^ elucidated the coupled excitation mechanism of Rotor-Stator Interaction (RSI) on pressure pulsation, vibration, and noise in a multistage pump through combined FSI numerical and experimental analysis. However, existing studies mostly focus on single-stage pumps or simplified models under conventional operating conditions. There is a lack of systematic research on the extreme conditions of deep-sea drilling fluid lift pumps, as well as on the vibration propagation and evolution characteristics associated with their multistage structure, particularly regarding the assessment and identification of multi-fault coupling risks, which remains to be further explored.

Regarding various faults caused by vibration in centrifugal pumps, scholars have focused on developing advanced monitoring and diagnosis methods, conducting in-depth research^39–41^. The existing fault diagnosis methods mainly include time-domain statistical indicators, spectrum analysis, time-frequency methods, and data-driven diagnosis based on vibration signals^42–44^. However, existing research predominantly focuses on single-stage pumps or simplified models, lacking systematic analysis of the complex structure of multistage pumps^45^. Furthermore, vibration testing experiments are typically conducted around the design flow rate (Qd), with insufficient attention paid to vibration evolution characteristics under off-design flow rates, leading to gaps in fault warning^46^. Conventional intelligent diagnostic methods mostly target single-fault classification, lacking dynamic assessment of the coupling effects of multiple faults, such as mechanical looseness, misalignment, and cavitation^47^. Few studies directly correlate vibration parameters with fluid excitation forces. Some deep learning-based models are computationally complex and time-consuming to train, making it difficult to meet the real-time monitoring requirements of deep-sea drilling^48,49^. Moreover, there is a lack of simple diagnostic indicators combined with field-measurable parameters.

To address the limitations of simplified pump models, insufficient off-design condition coverage, and limited engineering applicability in existing studies, this work investigates the vibration evolution and fault risk of a six-stage deep-sea drilling fluid lift pump by integrating CFD simulation, FSI-based vibration analysis, prestressed modal analysis, and full-flow-range experiments. The internal flow characteristics and fluid excitation under rated conditions are first obtained through CFD, and the structural dynamic response of the pump is evaluated using prestressed modal analysis. Subsequently, performance and vibration tests are conducted over the full flow range of 0–260 m³/h to validate the numerical model and examine vibration behavior under rated and off-design conditions. Based on the RMS of vibration velocity, the Crest Factor of acceleration, and time-domain waveform characteristics, a practical fault-risk evaluation framework is established to identify potential vibration-related faults, such as mechanical looseness and misalignment. Finally, the optimal operating range of the lift pump is determined, providing engineering guidance for vibration control, fault warning, and safe operation of multistage centrifugal pumps in deep-sea drilling applications. This research can guide the formulation of operational strategies, such as avoiding high-vibration risk intervals and pre-identifying potential fault modes (e.g., mechanical looseness, misalignment). Ultimately, the application of this methodology aims to reduce unplanned failure risks and the extension of equipment lifecycle, even in complex environments like deep-sea drilling, after necessary calibrations for site-specific conditions (e.g., pressure, fluid properties).

## Numerical simulation of RMR system and lifting pump

### RMR system composition and working principle

**Fig. 1:**
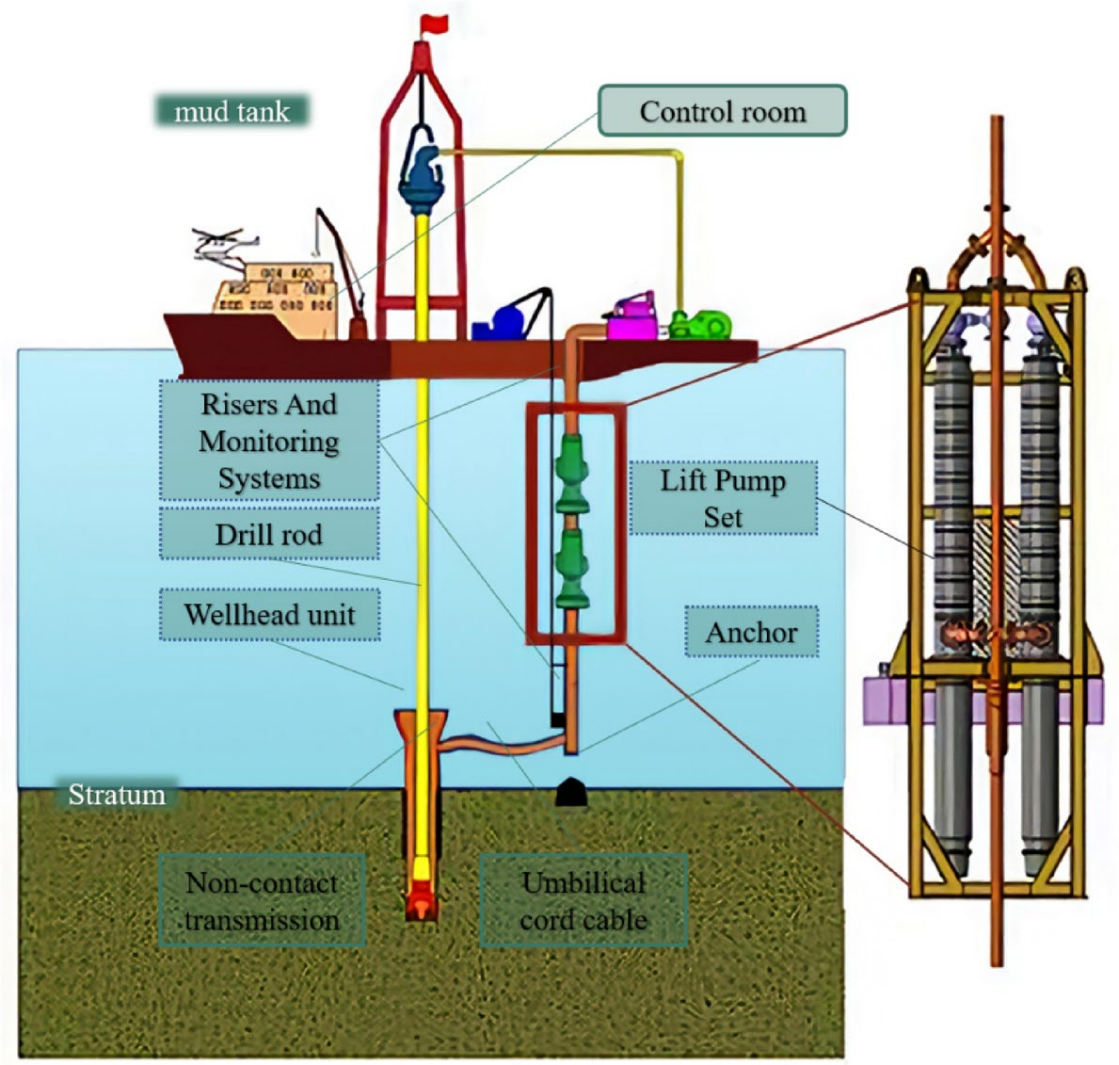
Overall scheme design of RMR system.

The Riserless Mud Recovery (RMR) system, as a representative deep-sea drilling equipment, consists of several core components, including the drilling platform, drill string, wellhead equipment, subsea lift pump unit, integrated umbilical cable, anchoring system, riser, and monitoring network. These modules operate synergistically to form a closed-loop drilling fluid circulation system. The overall system schematic is illustrated in Fig. 1. The subsea lift pump unit is a critical piece of equipment based on a multistage centrifugal pump design. With a single-stage head of up to 56 m, it can effectively counter hydrostatic pressure at water depths of several thousand meters. Regarding the operational mechanism, the RMR system relies on subsea pump lift technology to achieve efficient drilling fluid recovery through negative pressure suction and multistage pressure boosting. During the drilling process, drilling fluid carrying cuttings returns through the wellbore annulus to the subsea mud suction module. It is then drawn in by the lift pump assembly and enters the pressurization stage. The dual-pump lift system adapts to different working conditions by switching the manifold through valve operation. The monitoring system analyzes the cuttings concentration and return fluid pressure in real-time, automatically optimizing the operating mode to achieve the intelligent lifting with pressure-on-demand and flow-on-demand.

### Establishment of simulation models

This study employs a six-stage centrifugal lift pump as the research object. The simulation model establishment process primarily includes four steps: geometric model creation, fluid domain construction, model simplification, and mesh generation. First, the internal flow channel model of the pump is extracted through Boolean operations, simplifying the single-stage pump flow channel while retaining the continuous fluid regions of the impeller and guide vanes. The guide vanes and impeller are separated to clearly simulate their interaction. Subsequently, the simplified single-stage flow channels are assembled into a complete six-stage model. For the solid domain model, geometric characteristics with minimal impact on overall stiffness and vibration modes are removed to ensure the accuracy of structural mechanical responses, retaining only key load-bearing components, such as the pump shaft, impeller, guide vanes, and flange connections.

**Fig. 2:**
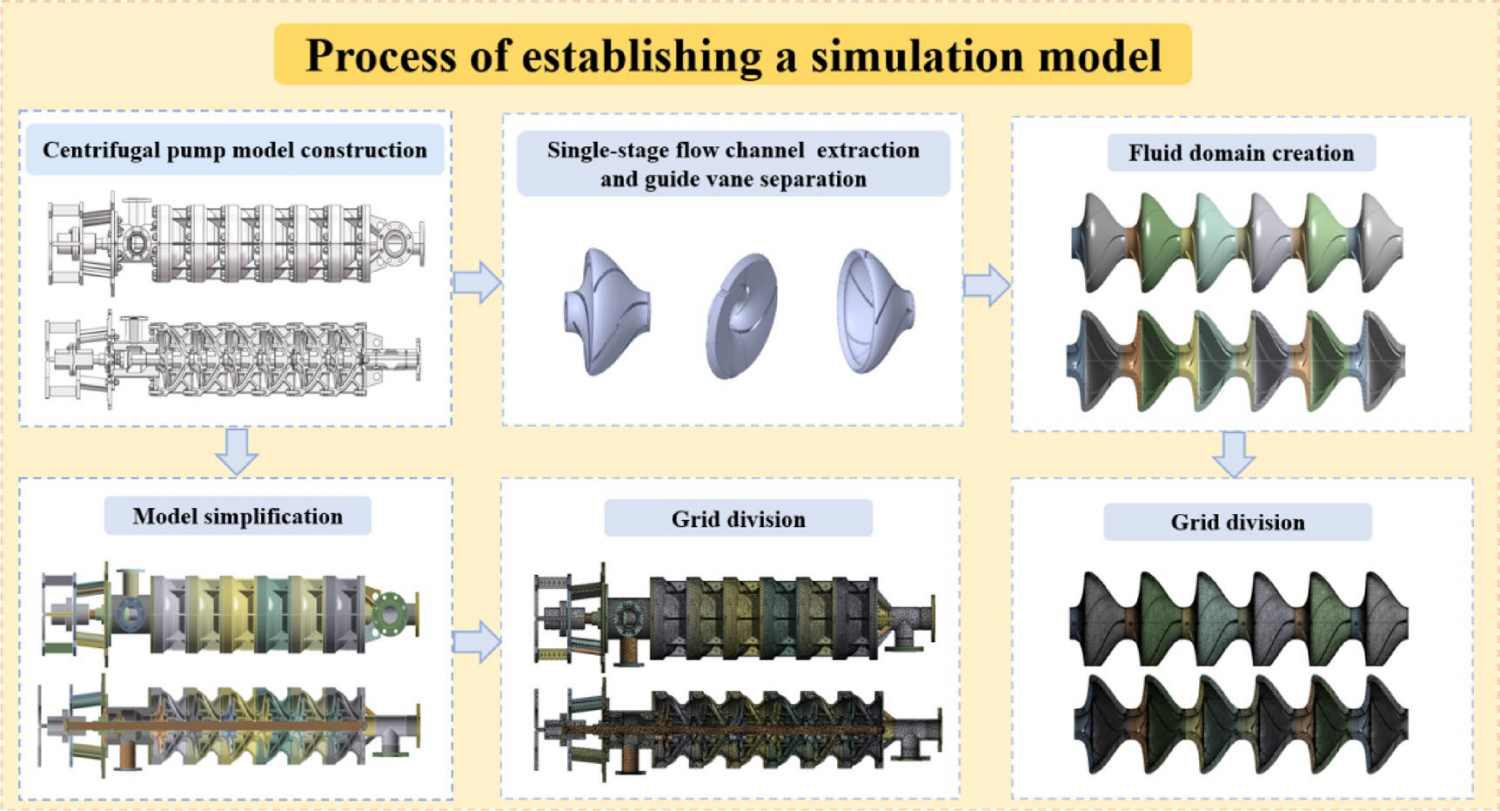
Simulation model establishment process.

After completing the simplification of the geometric model, meshing was performed separately for the fluid domain and the solid domain. The ANSYS Meshing platform was employed, utilizing unstructured tetrahedral grids with local refinement to accommodate complex geometries, such as impeller curved surfaces and narrow flow passages, reducing grid distortion while accurately capturing high-gradient flow characteristics. Grid independence verification shows that the head tends to stabilize when the total number of cells reaches approximately 890,000 and 1,470,000, respectively. The average mesh element quality is above 0.81 and 0.75, respectively. Figure 2 illustrates the systematic workflow for establishing the simulation model.

### Numerical simulation methods and boundary conditions

In the present study, the internal flow was assumed to be a three-dimensional, incompressible, single-phase Newtonian turbulent flow with constant fluid properties. Heat transfer, phase change, and compressibility effects were neglected. The governing equations for the fluid are the incompressible Reynolds-Averaged Navier-Stokes (RANS) equations. The core algorithm of equations includes:

Continuity equation:

1 $$\nabla \cdot \vec {u}=0$$

Where $\nabla \cdot \vec {u}$ represents the divergence of the velocity field (units: s⁻¹), indicating the net outflow rate of fluid per unit volume, and $\vec {u}$ is the fluid velocity vector (units: m/s), describing the magnitude and direction of the fluid motion at any point in space.

Momentum equation:

2 $$\rho \left( {\frac{{\partial \vec {u}}}{{\partial t}}+\vec {u} \cdot \nabla \vec {u}} \right)= - \nabla p+\mu {\nabla ^2}\vec {u}+\rho \vec {f}$$

Where $\rho$ is the fluid density (units: kg/m³); $\frac{{\partial \vec {u}}}{{\partial t}}$ is the local acceleration term (units: m/s²), representing the rate of velocity change over time. $\vec {u} \cdot \nabla \vec {u}$ is the convective acceleration term, describing acceleration due to the spatial motion of the fluid. $\nabla p$ is the pressure gradient (units: Pa/m), acting as the primary driving force for fluid flow. $\mu$ is the dynamic viscosity of the fluid (units: Pa·s). ${\nabla ^2}\vec {u}$ is the Laplacian of the velocity, representing the viscous diffusion effect. $\rho \vec {f}$ is the body force per unit volume (units: N/m³), where $\vec {f}={\mathrm{g}}= - 9.81{\mathrm{m}}/{{\mathrm{s}}^{\mathrm{2}}}$ (acceleration due to gravity).

The Shear Stress Transport (SST) k-ω turbulence model was selected for its capability to accurately capture near-wall flow behavior and shear layer characteristics, making it well-suited for simulating complex vortices and separated flows in centrifugal pumps. The model equations are defined as follows.

The turbulent kinetic energy (k) transport equation:

3 $$\frac{{\partial (\rho k)}}{{\partial t}}+\nabla \cdot (\rho k\vec {u})=\nabla \cdot \left[ {\left( {\mu +\frac{{{\mu _t}}}{{{\sigma _k}}}} \right)\nabla k} \right]+{P_k} - {\beta ^*}\rho k\omega$$

Where $k$ is the turbulent kinetic energy (units: m²/s²), representing the time-averaged kinetic energy of turbulent fluctuations; ${\mu _t}$ is the turbulent viscosity (units: Pa·s), used to model turbulent stresses. ${\sigma _k}$ is the turbulent Prandtl number, a dimensionless constant (typically taken as 1.0). ${P_k}$ is the production term of turbulent kinetic energy (units: kg/(m·s³)), generated by mean flow shear. ${\beta ^*}$is a model constant (typically 0.09), controlling the turbulence dissipation rate. $\omega$ is the specific dissipation rate (units: s⁻¹), representing the rate at which turbulent kinetic energy is dissipated.

The specific dissipation rate (ω) transport equation:

4 $$\frac{{\partial (\rho \omega )}}{{\partial t}}+\nabla \cdot (\rho \omega \vec {u})=\nabla \cdot \left[ {\left( {\mu +\frac{{{\mu _t}}}{{{\sigma _\omega }}}} \right)\nabla \omega } \right]+\frac{\gamma }{{{\nu _t}}}{P_k} - \beta \rho {\omega ^2}+2(1 - {F_1})\rho \frac{1}{{{\sigma _{\omega ,2}}}}\nabla k \cdot \nabla \omega$$

Where ${\nu _t}$ is the kinematic turbulent viscosity (units: m²/s), defined as ${\nu _t}={\mu _\tau }/\rho$; ${\sigma _\omega }$ is the turbulent Prandtl number, a dimensionless constant. $\gamma$ is a model coefficient. $\beta$ is the dissipation term constant, typically considered as 0.075; ${F_1}$ is a blending function, with a value of 1 near walls and 0 in regions far from walls. ${\sigma _{\omega ,2}}$ is an auxiliary constant for the cross-diffusion term.

The Computational Fluid Dynamics (CFD) numerical simulation of the centrifugal pump fluid domain was conducted using ANSYS Fluent 2022 R1. In the present study, clean water at 20 °C was used in both the numerical simulation and laboratory experiment as a simplified working medium to ensure consistency between model validation and controlled vibration testing. Therefore, the Newtonian fluid assumption was adopted to capture the baseline hydraulic loading and vibration evolution of the pump under simplified conditions. The impeller region was defined within a rotating reference frame with a rated speed of 1450 rpm, while the remaining regions were designated as stationary fluid domains. Gravitational effects were accounted by setting the gravitational acceleration vector to *g* = − 9.81 m/s ^2^. For the boundary conditions, the inlet was specified as a pressure inlet with a total pressure of 0.101 MPa (gauge), derived from experimental data measured at the pump rated flow condition. The outlet boundary condition was set to a mass flow outlet, with the value corresponding to the rated volumetric flow rate of 126 m³/h. All walls in the impeller region were set as rotating walls, while the other pump walls were treated as stationary no-slip walls. The interface between rotating and stationary regions was handled using the Multiple Reference Frame (MRF) method. The pressure-based coupled algorithm was selected as the solver, employing second-order upwind discretization schemes. The solution was considered converged when the residuals fell below the prescribed thresholds. The specific simulation parameters are summarized in Table 1.

**Table 1:** Simulation parameter settings.

Name	Numerical value
Density (kg/m³)	998.2
Viscosity (mPa·s)	1.003
Inlet total pressure (Mp)	0.101
Outlet rated flow(m³/h)	126
Rated speed (rpm)	1450

Prestressed modal analysis was conducted based on flow field calculations to evaluate the natural vibration characteristics of the six-stage centrifugal pump under actual operating conditions, specifically under steady-state fluid and gravitational loads. The pump structure was assumed to be homogeneous, isotropic, and linearly elastic. The modal analysis was performed under the small-deformation assumption, and the hydraulic load transferred from the CFD results was treated as a static prestress. The analysis employed a sequential coupled simulation approach. First, the steady-state fluid pressure field obtained from the Fluent calculation, representing the rated operating condition, was applied as a pre-stress load onto the structural model of the pump. A static structural analysis was then performed to calculate the stress distribution and changes in structural stiffness of the pump body under the combined action of fluid pressure, gravity, and rotational centrifugal forces. In this prestressed condition, an eigenvalue problem was addressed to calculate the first several natural frequencies and their corresponding mode shapes of the pump structure. The governing equation is the eigenvalue problem that includes prestress:

5 $$\left( {[K]+[S]} \right)\{ \phi \} ={\omega ^2}[M]\{ \phi \}$$

Where $[K]$ is the stiffness matrix (units: N/m), describing the structure resistance to deformation; $[S]$ is the stress stiffness matrix (units: N/m), generated by the pre-stress (such as fluid pressure and centrifugal forces); $[M]$ is the mass matrix (units: kg), representing the inertial characteristics of the structure; $\omega$ is the angular frequency (units: rad/s); $\{ \phi \}$ is the mode shape vector (dimensionless), describing the vibrational pattern of the structure at a specific frequency.

**Fig. 3:**
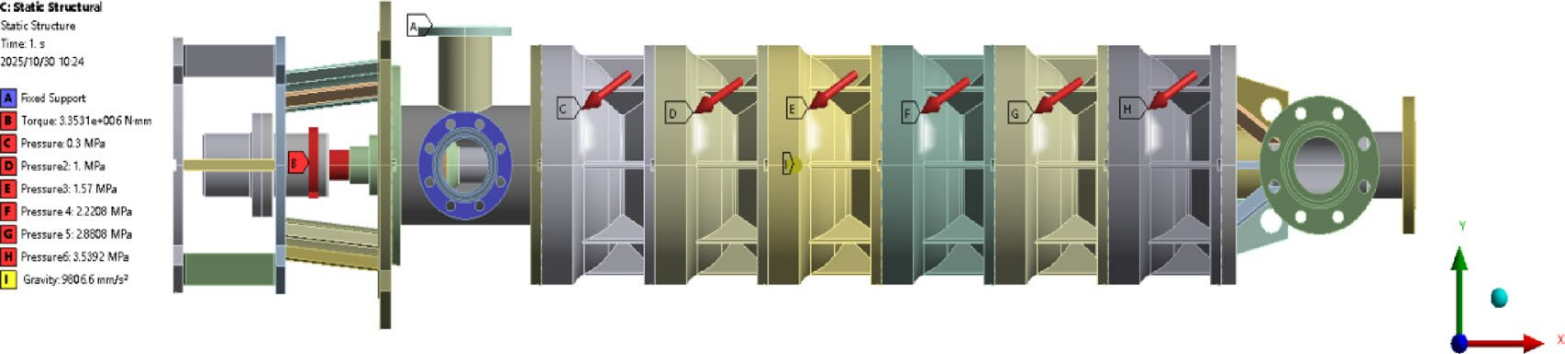
Constraints and loads.

The main structural components of the simplified six-stage centrifugal pump (including the connecting section, suction section, pump shaft, impellers, guide casing, and outlet connector) were constructed from 2205 duplex stainless steel. The material parameters included the elastic modulus and Poisson ratio. The shaft sleeve was constructed from YG8 hard alloy to enhance wear resistance. In the boundary condition setup, the flange connection surfaces where the inlet and outlet of the pump connect to the piping, as well as the pump shaft connected to the motor, were set as fixed constraints. It should be noted that the fixed-constraint treatment adopted at the flange connections is an idealized simplification. In actual subsea deployment, the lift pump is coupled with flexible umbilicals and supporting structural frames, which may provide more compliant boundary conditions than perfectly rigid supports. Therefore, the present assumption may increase the effective structural stiffness of the model and lead to some overestimation of the natural frequencies. Nevertheless, this simplification remains useful for identifying the primary modal characteristics and evaluating the relative resonance safety margin of the pump structure. The pressures on each stage of the impeller and the torque obtained from the internal flow field simulation in Fluent were applied as external loads to the impellers and shaft of the pump, respectively. The gravitational acceleration was set, acting in the negative X-direction. Figure 3 shows the schematic diagram of constraints and loads for the six-stage pump.

### H-Q curve validation between simulation and experiment

The head-flow (H-Q) curve serves as a core performance indicator representing the energy conversion efficiency of the centrifugal pump. To systematically evaluate the H-Q characteristics of the six-stage centrifugal pump and validate the accuracy of the simulation model, a comparative analysis between performance testing and numerical simulation was conducted at the rated speed of 1450 rpm. Based on the established vibration test platform, different flow conditions were simulated by adjusting the pipeline valve opening (sequentially set to 5.5%, 20.7%, 23.5%, 23.6%, 24.7%, 24.8%, 27%, 27.4%, 28.2%, and 30%). An electromagnetic flowmeter and pressure sensors were employed to collect flow rate and inlet/outlet pressure data of the pump at each valve opening. In the numerical simulations, the experimentally measured inlet pressure and outlet flow rate were used as boundary conditions for the Fluent simulation to obtain the corresponding simulated pressure values.

The pump head, defined as the energy increment per unit weight of liquid passing through the pump, was calculated. Since the inlet and outlet pipeline diameters remained consistent and pipeline losses were neglected, the head was determined using the following formula:

6 $$H=\frac{{{p_{{\mathrm{out}}}} - {p_{{\mathrm{in}}}}}}{{\rho g}}$$

where *p*_*out*_ is the pump outlet pressure (Pa), *p*_*in*_ is the pump inlet pressure (Pa), *ρ* is the fluid density (kg/m³, taken as 998.2 kg/m³ for clean water), and *g* is the gravitational acceleration (9.81 m/s²).

**Fig. 4:**
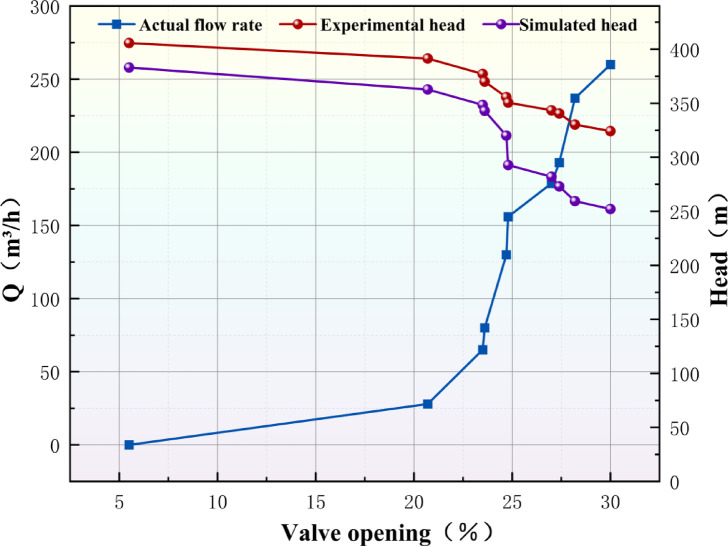
H-Q Performance curve comparison.

A comparison of the H-Q performance curves obtained from experiments and simulations across the valve opening range of 5.5–30% is presented in Fig. 4.

Figure 4 demonstrates a high degree of consistency in the variation trend between the simulated and experimental head-flow (H-Q) curves across the entire valve opening range of 5–30%. Both curves exhibit a smooth decrease in head with increasing flow rate, and the head values at various flow transition points show high consistency. This indicates that the simulation model successfully captures the core physical mechanism of energy conversion within the pump. Although the simulated head values are slightly lower than the experimental data throughout the range, indicating a consistent systematic deviation, this bias remains stable. The accurate replication of the curve morphology confirms that the simulation reliably captures the essential characteristics of the flow field. The observed discrepancies are primarily attributed to minor, calibratable differences in the modeling of hydraulic losses, which do not undermine the capability of the model to predict performance trends. At the rated flow condition (Q = 126 m³/h), the simulated head (329 m) and the experimental head (354 m) both are close to the theoretical head of 336 m. These results confirm the reliability and accuracy of the simulation model for the six-stage lift pump against experimental outcomes.

## Numerical simulation result analysis and experimental testing of the lift pump

### Numerical simulation result analysis of the lifting pump

**Fig. 5:**
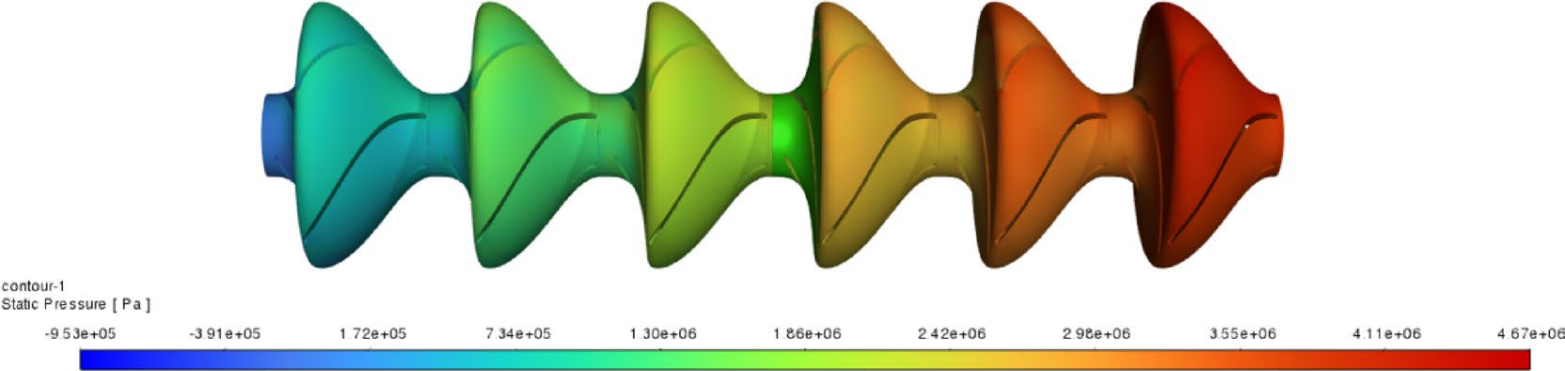
Static pressure distribution contour.

**Fig. 6:**
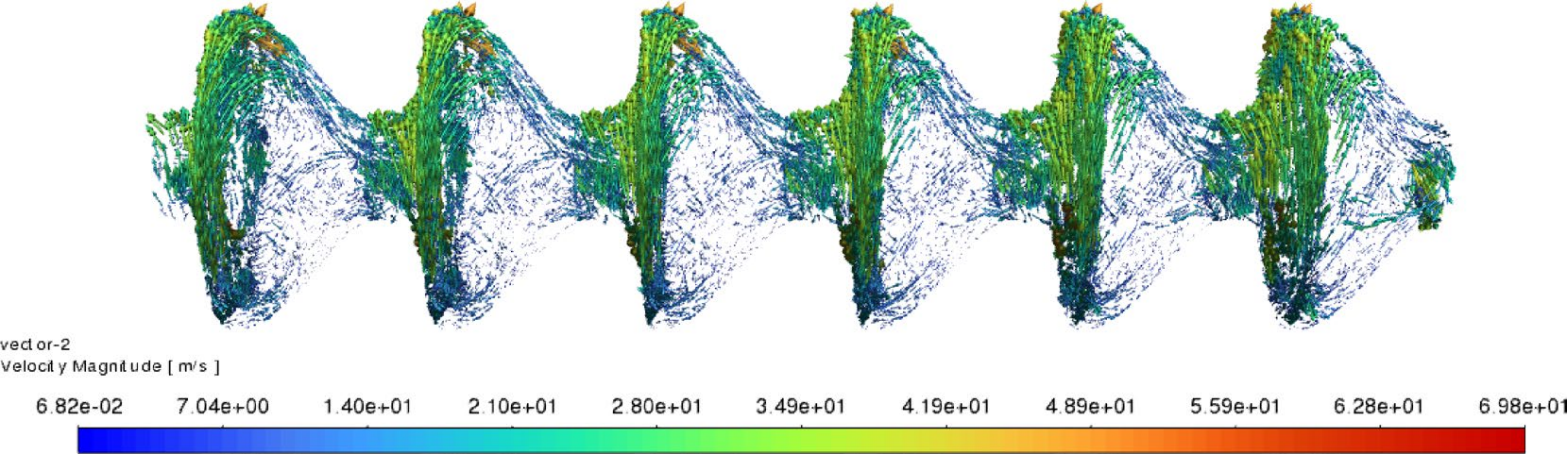
Velocity vector plot.

Figures 5 and 6 respectively illustrate the static pressure distribution and velocity vector distribution within the six-stage centrifugal pump under rated operating conditions (speed: 1450 rpm, flow rate: 126 m³/h). As revealed by the static pressure distribution contour, the pressure demonstrates a uniform and continuous gradient increase from the inlet of the first-stage impeller to the outlet of the final-stage impeller. The pressure increase across each individual impeller stage is essentially consistent. Within a single impeller, the pressure distribution is largely axisymmetric. This symmetry indicates that the radial fluid forces on the rotor are relatively low. These observations collectively indicate stable operation of the pump and highly efficient internal flow under the rated condition. The velocity vector plot indicates that at the rated flow rate, the velocity vectors in the main flow passages are directionally aligned, and the streamlines are smooth. There are no significant flow separation, backflow, or significant vortex structures, confirming that the pump operates with high efficiency close to its design point and that the overall flow condition is favorable. However, despite the overall stable flow, minor vortices or jet-wake structures persist in localized regions, such as at the junction between the pressure and suction sides of the impeller blades and near the guide vane heads. These localized, small-scale unsteady flow phenomena could be the cause of pressure pulsations at high frequencies.

**Fig. 7:**
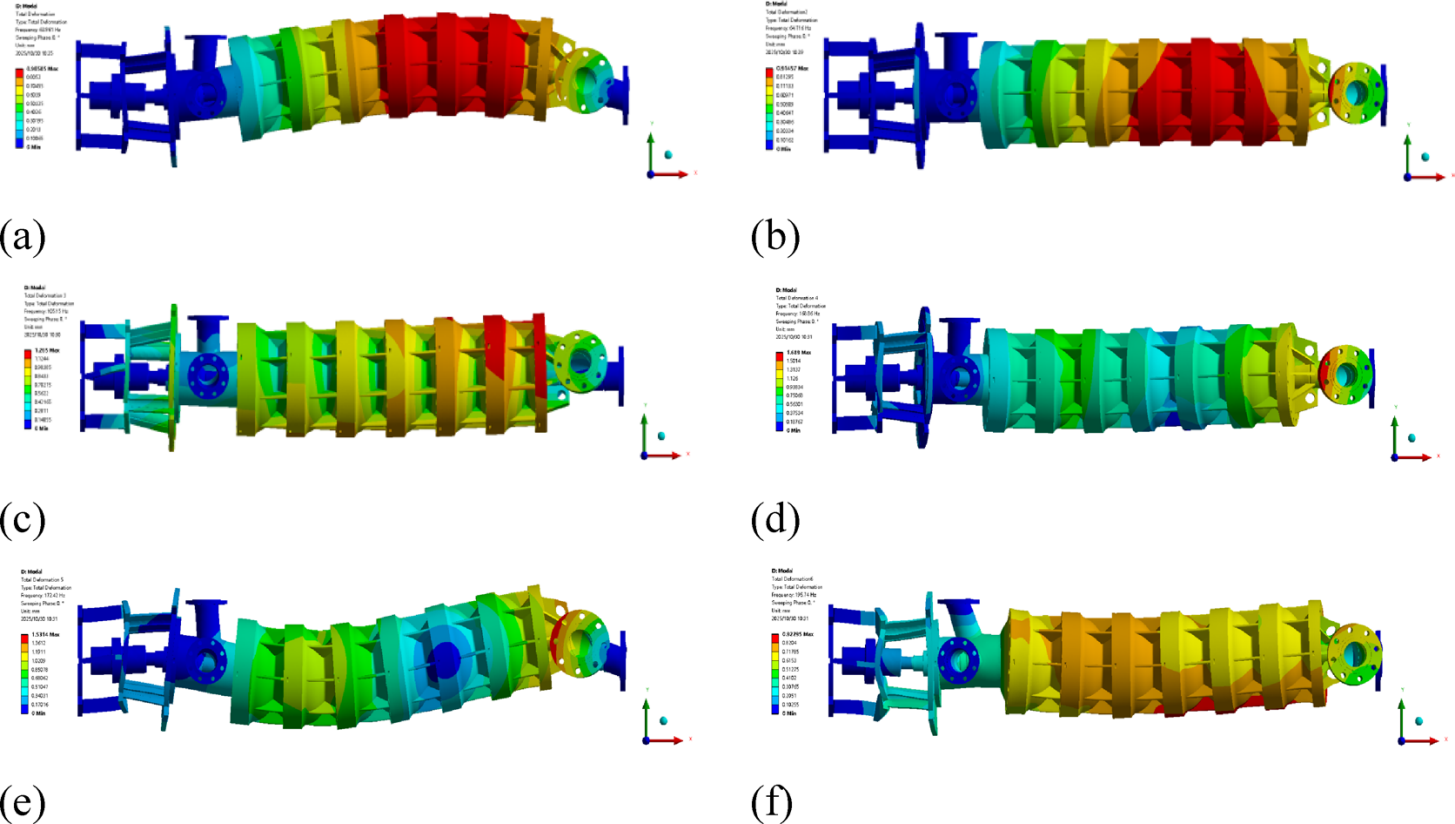
The first six vibration modes of a six-stage centrifugal pump. (**a**) First-order mode shape, (**b**) Second-order mode shape, (**c**) Third-order mode shape, (**d**) Fourth-order mode shape, (**e**) Fifth-order mode shape and (**f**) Sixth-order mode shape.

Based on the prestressed modal analysis, the first six natural frequencies and corresponding mode shapes of the six-stage centrifugal pump under prestressed conditions were successfully extracted. These mode shapes visually represent the relative magnitude and distribution of vibrational displacement in the pump structure at different frequencies, as illustrated in Fig. 7.

The mode shapes of the centrifugal pump primarily include swinging vibration, torsional vibration, bending vibration, and pitching vibration. Table 2 summarizes the first six natural frequencies, along with their mode shape characteristics and primary directions.

**Table 2:** First six natural frequencies and mode shape characteristics.

Mode order	Natural frequency (Hz)	Mode shape characteristic	Directions
1	63.961	Swinging Vibration	Y
2	64.716	Swinging Vibration	Z
3	105.15	Torsional Vibration	YZ Plane
4	168.86	S-shaped Bending Vibration	XZ Plane
5	172.42	S-shaped Bending Vibration	XY Plane
6	195.74	Pitching Vibration	X

In centrifugal pumps, the excitation forces primarily originate from periodic forces generated by rotating components (e.g., shaft and impellers of the pump). Their frequencies are related to the shaft rotational frequency (shaft frequency) or the Blade Pass Frequency (BPF). The shaft frequency is the number of rotations per second of the pump shaft, related to the rotational speed (rpm). The BPF is the number of times the blades pass a fixed point on the pump casing per second, calculated using the following formula:

7 $${f_{bpf}}=Z \times \frac{{\mathrm{n}}}{{60}}$$

where *Z* is the number of blades (here, *Z* = 3), and *n* is the rotational speed of the impeller (units: rpm).

**Fig. 8:**
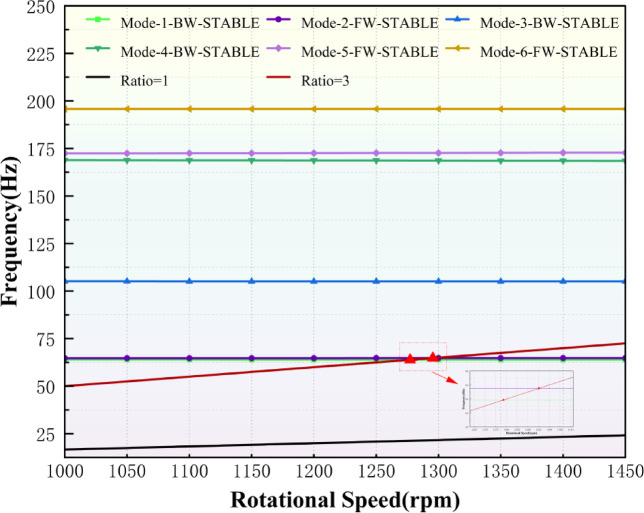
Campbell diagram.

The Campbell diagram is shown in Fig. 8. The sloping lines originating from the origin represent the excitation frequencies (for ratios of 1 and 3, corresponding to shaft frequency and BPF, respectively), while the horizontal lines represent the natural frequencies. Intersections between these lines indicate potential resonance conditions. Analysis of Fig. 8 reveals that the first-order and second-order critical speeds of the six-stage centrifugal pump are 1278.4 rpm and 1295.2 rpm, respectively. Both are below the rated speed of 1450 rpm, indicating potential resonance during startup process. It is crucial to rapidly traverse this speed range during startup and avoid prolonged operation at these speeds. Conversely, the excitation frequencies at the rated speed (1450 rpm), specifically the fundamental frequency of 24.17 Hz and its harmonics, maintain a sufficient safety margin from the first six natural frequencies of the pump (with the lowest at 63.96 Hz). This theoretically avoids resonance at the rated operating point, confirming it as a safe operating condition.

### Test platform setup and measuring point arrangement

On the premise that the lift pump operates stably without resonating under the rated operating condition, indoor vibration tests were performed to investigate the vibration characteristics of the pump under different flow conditions. The experiments were conducted on the actual pump prototype rather than on a geometrically scaled model. Consequently, strict scaling laws based on the Reynolds, Euler, or Strouhal numbers were not involved. Instead, the transferability of the results is mainly supported by the consistency of pump geometry, rotational speed, and hydraulic operating regime.

**Fig. 9:**
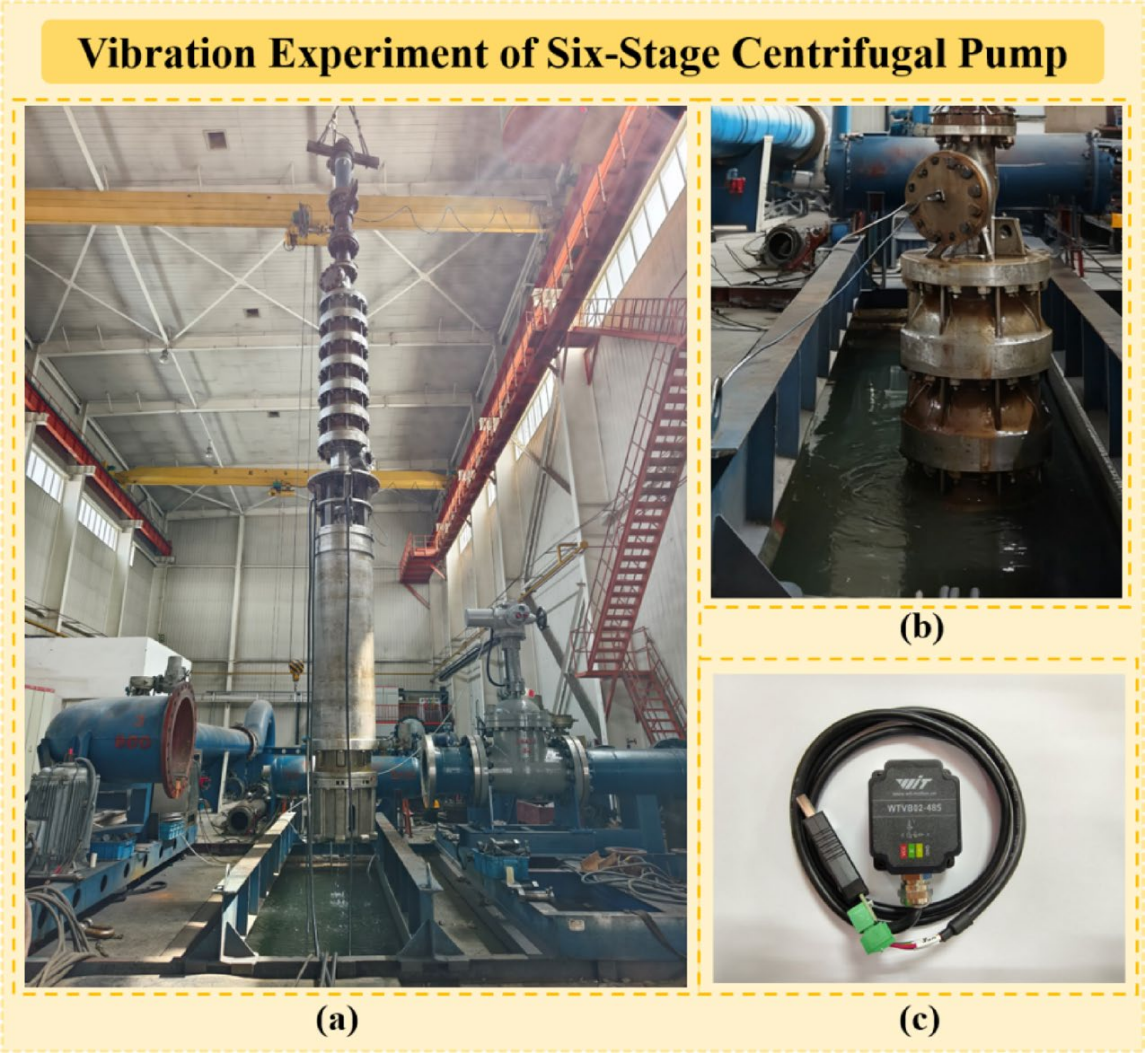
Test platform composition. (**a**) Test layout, (**b**) Vibration measurement points and (**c**) Vibration sensors.

The present experiments are intended to provide a baseline assessment of pump vibration behavior and fault evolution under simplified and well-controlled conditions, while the effects of deep-sea hydrostatic pressure, low temperature, and realistic drilling-fluid properties were not included. Figure 9 shows the configuration of the vibration test platform, including the overall layout, the measuring point arrangement, and the installation of the vibration sensors. The tests were conducted in a standard laboratory using clean water as the working medium, with the ambient temperature maintained at approximately 20 °C to reduce external environmental interference. The test platform mainly consisted of the following parts: (1) the six-stage centrifugal lift pump and driving motor connected through a coupling; (2) the inlet and outlet pipelines, valves, and pressure gauges used for flow regulation and operating-condition monitoring; (3) the steel support base fixed to the floor to reduce external vibration transmission; and (4) the data acquisition and control unit for real-time recording of vibration signals.

To obtain representative vibration responses from the pump structure, the vibration measuring point was located at the outlet flange of the pump, where the structural response is relatively sensitive and the vibration energy is more concentrated. In addition, this measurement point is directly connected to the main hydraulic load transfer path, enabling it to reflect the coupled vibration response arising from the combined action of internal fluid excitation and structural transmission. Although minor local fault characteristics are typically more sensitive at the bearing housing, this study focuses on the overall vibration evolution and fault risk characteristics of the pump unit, while acknowledging that signal attenuation caused by housing mass is a limitation of the current measurement scheme. A digital triaxial vibration sensor (model: WT-VB02-485) was rigidly mounted on the outer surface of the outlet flange. The measurement directions were defined according to the pump coordinate system: the X-direction denotes the radial horizontal direction, the Y-direction denotes the axial direction along the shaft, and the Z-direction denotes the radial vertical direction. In this way, the vibration responses in two radial directions and one axial direction were measured simultaneously at the same structural location. During installation, the sensor was securely mounted to the measurement point to ensure reliable transmission of structural vibrations and avoid signal distortion caused by mounting looseness. Before each test, the sensor orientation was checked to ensure consistency between the physical installation direction and the defined coordinate system. The sensor cable and acquisition interface were also secured to minimize interference during pump operation. Before formal data acquisition, the vibration sensor and the corresponding acquisition channel were inspected according to the instrument specifications. The inspection mainly included confirmation of axis orientation, zero-offset stability, and signal-output consistency, so as to ensure the reliability and repeatability of the vibration measurements. During the experiment, the vibration signals in the X, Y, and Z directions were recorded simultaneously under each flow condition for comparative analysis of directional vibration characteristics.

### Vibration measurement methods and evaluation criteria

Vibration testing serves as a core method for assessing the operational status of centrifugal pumps. Its foundation lies in quantifying vibration signals to identify potential faults and ensure equipment safety. In the pump industry, vibration severity is a critical standard for evaluating the operational stability of products. The assessment of pump’s vibration intensity primarily follows the international standard GB/T 29531-2013, “Measurement and Evaluation of Vibration in Pumps,” which utilizes vibration severity as the key evaluation parameter. Vibration severity is defined as the Root Mean Square (RMS) value of the vibration velocity signal, reflecting the average intensity of vibration energy. It is directly related to structural fatigue, noise, and the reliability of the pump. The vibration severity grade is determined by comparing measured values against standard thresholds, ultimately categorizing the vibration level of the pump into four classes: A, B, C, and D, as detailed in Table 3. In addition to RMS, the Crest Factor (Cf) of acceleration, typically ranging between 3 and 5, is employed as an auxiliary indicator in vibration analysis to capture transient impact characteristics. The threshold and classification criteria for Cf are presented in Table 4. In digital acquisition systems for vibration data, signals are discretized into N sampling points. The RMS value is calculated using the following formula:

7 $${v_{{\mathrm{rms}}}}=\sqrt {\frac{1}{N}\sum\limits_{{i=1}}^{N} {v_{i}^{2}} }$$

where *v*_*i*_ is the instantaneous vibration velocity (unit: mm/s) at the i-th sampling point, and *N* is the total number of samples.

The Crest Factor (Cf) of acceleration is defined as the ratio of the peak acceleration value to the RMS acceleration value, a dimensionless quantity calculated by:

8 $$Cf=\frac{{{a_{peak}}}}{{{a_{rms}}}}$$

where ${a_{peak}}$ is the peak value (absolute maximum) of the acceleration signal, and ${a_{rms}}$ is the RMS value of the acceleration (unit: m/s²).

In the present study, their combined use is not intended as a new standalone diagnostic index but as a practical joint evaluation framework for the six-stage deep-sea lift pump under full-flow operating conditions. Specifically, the RMS value is used to describe the overall vibration energy and severity level, while the Cf is used to identify transient impact characteristics embedded in the vibration response. A single indicator may not fully reflect the evolving fault state because off-design operating conditions often generate both continuous vibration enhancement and intermittent impulsive components. By combining RMS and Cf, the method provides a more comprehensive description of both global vibration deterioration and local impact-related abnormalities, thereby improving the interpretability of fault risk under different flow conditions.

However, the vibration measurements in this study are influenced by experimental uncertainty and data variability. Possible sources include limitations in sensor accuracy, differences in the installation and alignment of the triaxial vibration sensors, background environmental disturbance, minor fluctuations in pump speed and valve opening during operation, as well as errors in signal acquisition and discretization. In addition, vibration signals under off-design flow conditions may contain transient and intermittent components, leading to a certain degree of cycle-to-cycle variation even under nominally identical operating conditions. Therefore, the measured RMS and Cf values should be interpreted as representative indicators of vibration behavior under each condition, rather than exact invariant quantities.

**Table 3:** Vibration severity classification based on RMS values of velocity.

RMS value range (mm/s)	Severity grade	Fault risk description
RMS < 1.12	Grade A (Excellent)	Vibration is minimal, indicating smooth operation with no significant fault risk.
1.12 ≤ RMS < 2.8	Grade B (Good)	Vibration is acceptable Minor imbalance or flow disturbance may exist.
2.8 ≤ RMS < 4.5	Grade C (Require Attention)	Vibration is significant, suggesting potential faults.
RMS ≥ 4.5	Grade D(Unacceptable)	Vibration is severe, indicating high-risk faults.

**Table 4:** Fault Risk Classification Based on Acceleration Crest Factor (Cf).

Cf value range	Classification level	Fault risk description
Cf < 3.0	Excellent (Grade A)	Indicates negligible impact risk.
3.0 ≤ Cf < 4.0	Good (Grade B)	Suggests a minor risk of impact.
Cf ≥ 4.0	Risky (Grade C)	Signifies a substantial impact risk.

## Experiment-based analysis of vibration evolution and fault risk assessment

### Vibration characteristics under rated operating conditions

**Fig. 10:**
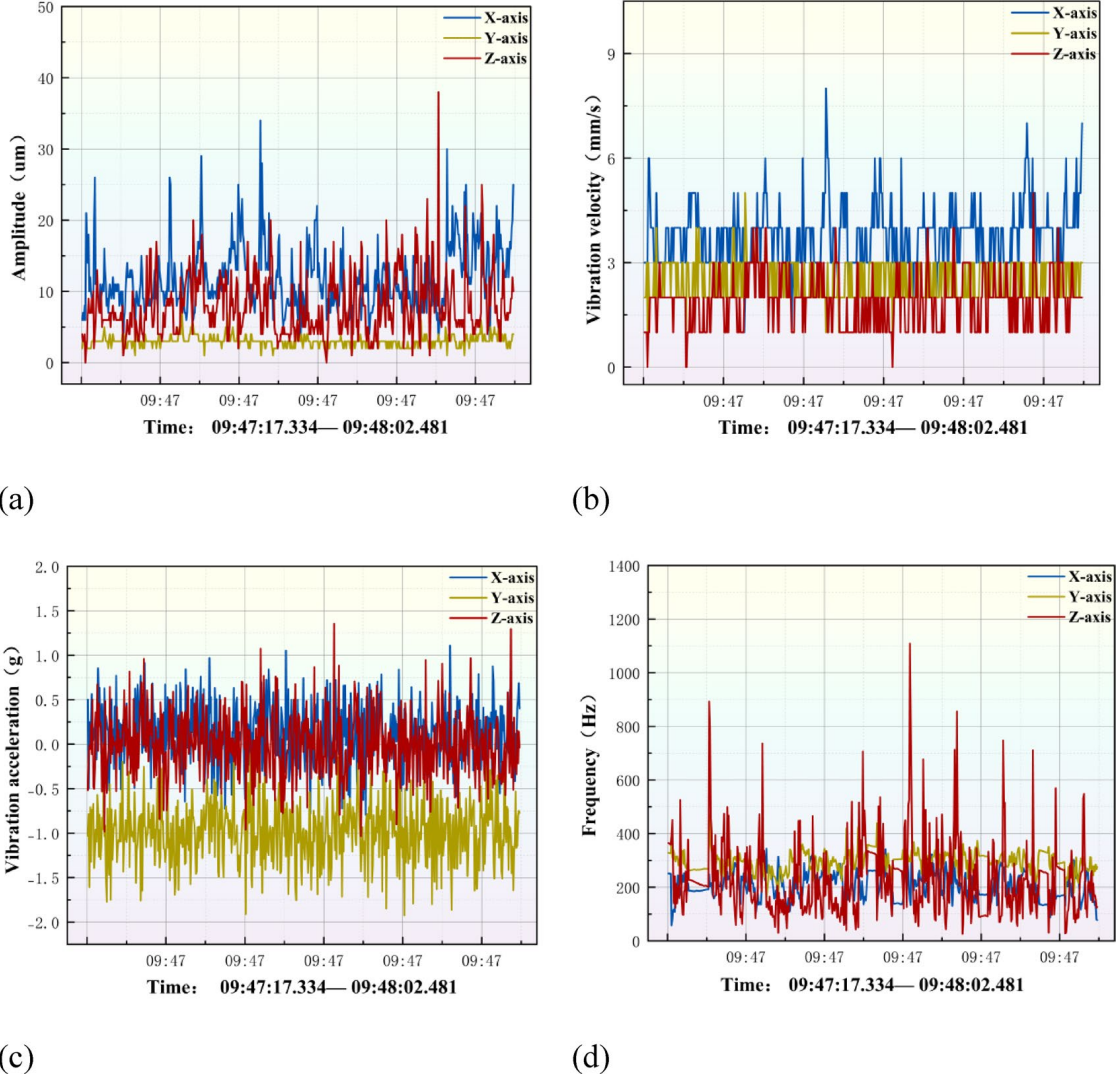
Time-domain response curve of vibration characteristics. (**a**) Amplitude, (**b**) Vibration velocity, (**c**) Vibration acceleration and (**d**) Vibration frequency.

Based on the vibration tests conducted on the lift pump, the time-domain waveforms of vibration responses, including amplitude, velocity, acceleration, and frequency, were captured at the outlet flange under rated operating conditions (rotational speed: 1450 rpm, flow rate: 126 m³/h). The results are illustrated in Fig. 10.

Based on the time-domain waveforms shown in Fig. 10, the maximum amplitudes and corresponding characteristic frequencies in the X, Y, and Z directions under rated conditions were established. The Root Mean Square (RMS) of vibration velocity and the Crest Factor (Cf) of acceleration were calculated, as summarized in Table 5.

**Table 5:** ​ Vibration parameter statistics of the centrifugal pump under rated conditions.

Direction	Max. amplitude (µm)	Characteristic freq. (Hz)	Velocity RMS (mm/s)	Crest factor of acceleration
X	34	65.2	3.896002	3.066331
Y	10	189.5	2.558798	1.841082
Z	38	83.1	2.041539	3.763455

By combining data analysis with industry standards, it can be concluded that under rated conditions, the pump’s vibration remains within safe thresholds, yet distinct characteristics are observed across different directions. The Y-axis (axial) demonstrates optimal performance with the lowest amplitude (10 μm) and favorable velocity RMS (2.56 mm/s) and Cf (1.84) values, indicating smooth harmonic motion. In contrast, the Z-axis (radial vertical) exhibits the highest amplitude (38 μm) and a Cf value of 3.76, suggesting minor transient impacts. Most importantly, the X-axis (radial horizontal) shows a potential resonance risk because its characteristic frequency (65.2 Hz) is close to the first two natural frequencies of the structure, making it the primary focus for vibration control despite its stable periodic waveform and acceptable RMS value (3.90 mm/s).

### Evolution of vibration characteristics under full flow conditions

**Fig. 11:**
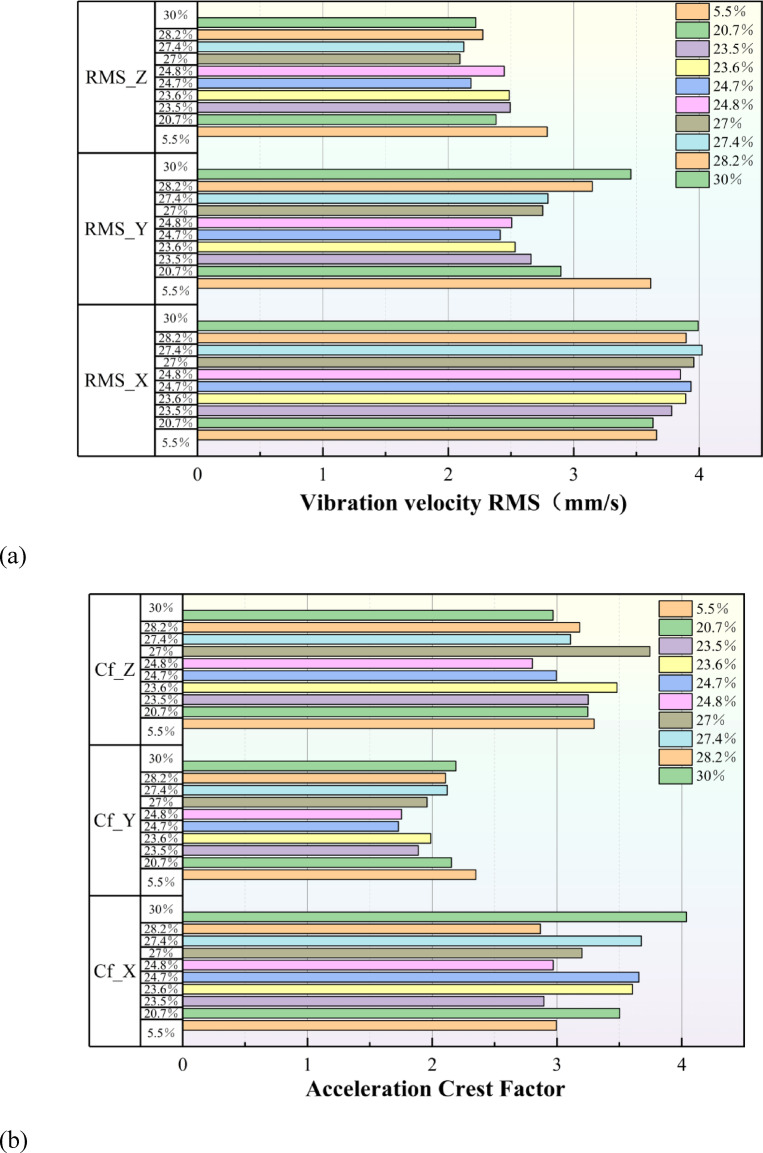
The RMS of vibration velocity and the Crest Factor (Cf) of acceleration under different valve openings. (**a**) The RMS of vibration velocity and (**b**) The Crest Factor (Cf) of acceleration.

To evaluate the influence of flow rate variations on the vibration characteristics of the centrifugal pump at rated rotational speed, vibration tests were conducted across the full flow range of 0–260 m³/h (corresponding to valve openings from 5.5 to 30%). The Root Mean Square (RMS) of vibration velocity and the Crest Factor (Cf) of acceleration along the X, Y, and Z axes under each flow condition were calculated to assess the dynamic response. The results are presented in Fig. 11.

**Fig. 12:**
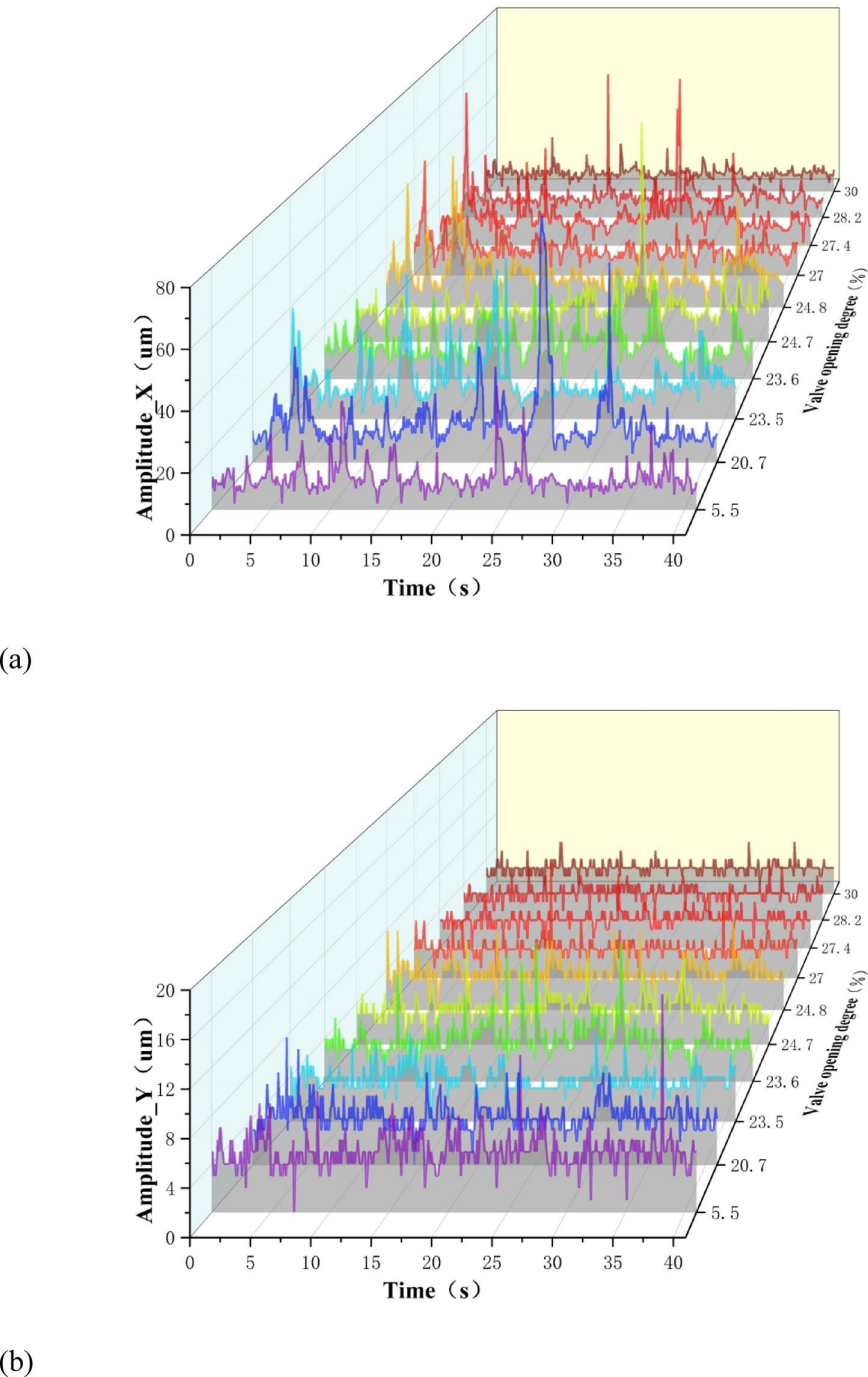

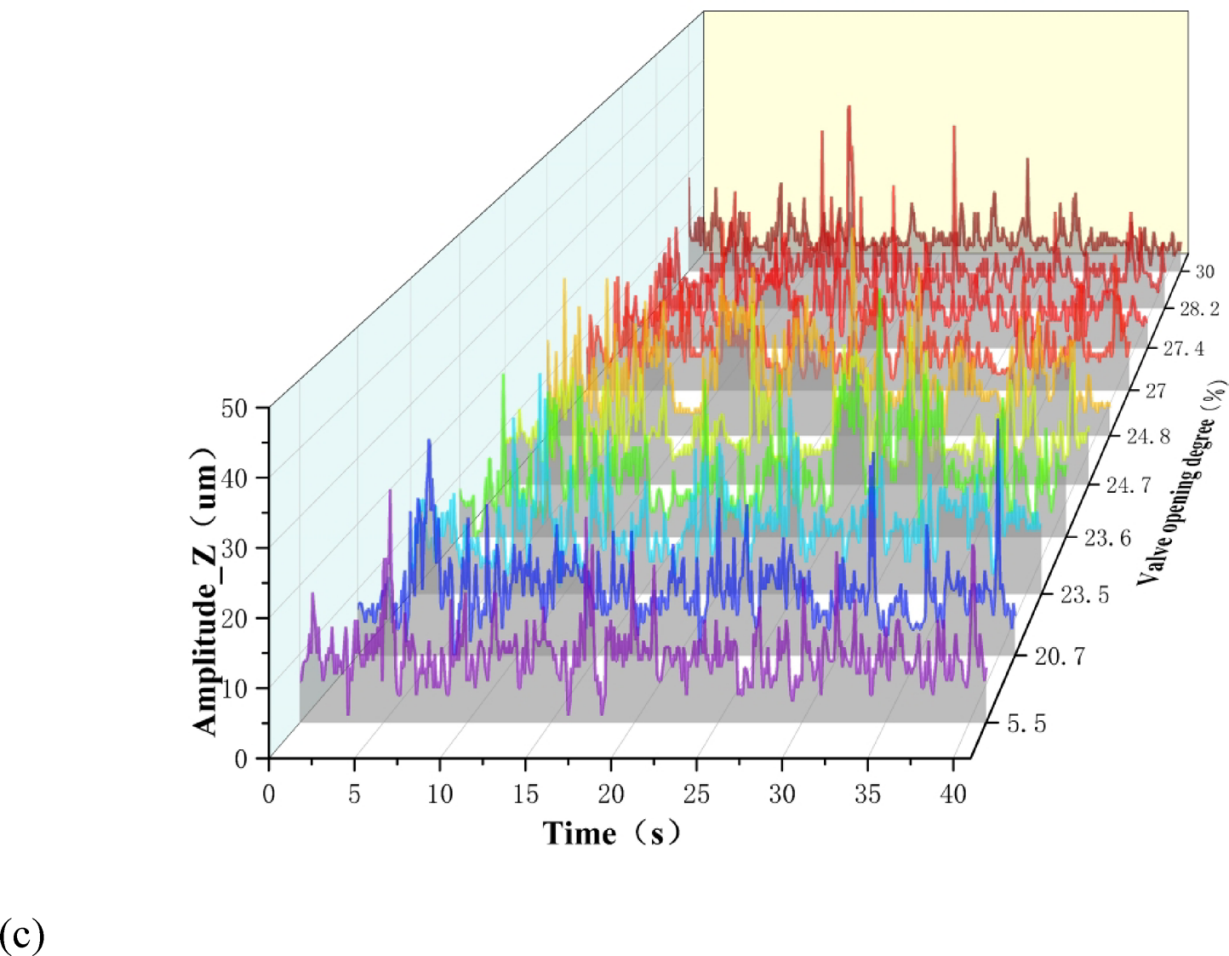
Time-domain amplitude response curves under different valve openings. (**a**) X-axis amplitude, (**b**) Y-axis amplitude and (**c**) Z-axis amplitude.

Meanwhile, the time-domain amplitude response curves of each axis within the same duration are presented in Fig. 12, allowing detailed analysis of waveform characteristics, such as transient impacts, periodicity, and modal excitations, under different operational conditions.

Based on the data presented in Fig. 11, it is observed that both the Root Mean Square (RMS) of vibration velocity and the Crest Factor (Cf) of acceleration show nonlinear progression with increasing valve opening. The RMS values represent the vibration energy intensity. The RMS values along the X-axis are generally high (ranging from 3.63 to 4.02 mm/s) and show a gradual increasing trend as the valve opening widens, indicating that the X-direction is relatively sensitive to flow variations. The Y-axis RMS reaches its lowest value (2.41 mm/s) at 24.7% valve opening, but increases at both low (5.5%) and high (30%) openings, indicating more stable vibration in the Y-direction near the design flow condition. The Z-axis RMS remains relatively stable (2.09–2.79 mm/s), with minor fluctuations.

The Cf values indicate the presence of impact-type vibration components. The X-axis Cf varies from 2.87 to 4.04, reaching the peak at 4.04 at 30% opening, which indicates a potential impact risk. The Y-axis Cf remains consistently low (1.73–2.35), demonstrating that vibration in this direction is predominantly smooth and steady. The Z-axis Cf falls within a moderate range (2.80–3.75), remaining below the 4.0 threshold. This indicates that vibration levels are acceptable, although potential fault risks still require attention.

Figure 12​ visually depicts the time-domain waveform characteristics of vibration amplitude along the X-, Y-, and Z-axes. At zero-flow (5.5% valve opening) and low-flow (20.7% valve opening) conditions, the X-direction (radial horizontal) waveform exhibits high-amplitude periodic pulses, often with high-frequency oscillations at the pulse peaks, demonstrating a typical “ringing” phenomenon. The pulse amplitude exceeds 80 μm, indicating the presence of strong periodic impact forces. In the Y-direction (axial), the waveform shows dense, irregular “sawtooth-shaped” peaks​ with significant amplitude fluctuations, indicating very frequent random impacts in the axial direction. The waveform characteristics in the Z-direction (radial vertical) are similar to those in the X-direction, exhibiting periodic pulses combined with high-frequency oscillations, but with a relatively lower amplitude (approximately 50 μm). When operating close to the rated flow condition (valve openings between 23.5 and 24.8%), the vibration characteristics exhibit systematic changes: the periodicity of the pulses in the X-direction​ becomes stronger, and the waveform becomes slightly more regular. However, the pulse amplitude remains high (58–85 μm), with persistent oscillations evident at the peaks. The “sawtooth” characteristic in the Y-direction​ diminishes significantly: the peaks in the waveform become relatively sparser and more uniform, and the amplitude decreases significantly to below 20 μm. This represents the most stable vibration stage for the Y-direction across all tested conditions, although the waveform still consists of discrete pulses rather than a smooth sinusoidal wave. The Z-direction​ continues to show periodic pulses with high-frequency oscillations, but the amplitude is reduced to 52 μm. Under high-flow conditions (valve openings between 27 and 30%), the vibration characteristics deteriorate further: the pulse amplitude in the X-direction​ increases, and the regularity of the pulse intervals is disrupted. The waveform encounters more irregular components and exhibits “beat vibration"​ phenomena (periodic amplitude modulation), indicating increasing complexity and strength of the external excitations. The Y-direction​ waveform becomes dense and irregular again, demonstrating that axial impact excitation intensifies with increasing flow. The Z-direction​ shows greater pulse amplitude, obvious intermittent peaks, and higher waveform disorder.

The correlation analysis between the time-domain amplitude response curves and the RMS/Cf data reveals that the vibration characteristics of the six-stage centrifugal pump show significant nonlinear evolution​ with varying flow rates. The increasingly nonlinear vibration behavior observed under off-design flow conditions is mainly caused by the combined effect of enhanced hydraulic excitation and structural response coupling. Near the rated flow condition, the internal flow remains relatively stable, with smooth streamlines and only small-scale local vortices near the impeller–guide vane interface. Under such conditions, the hydraulic load acting on the rotor–stator system is comparatively uniform, resulting in a relatively regular vibration response. However, when the flow rate deviates from the rated value, the internal flow field gradually deteriorates. Under low-flow conditions, flow separation, local recirculation, and vortex evolution are more likely to occur inside the impeller and guide vane passages, which can introduce unsteady radial force and intermittent pressure pulsation. Under high-flow conditions, increased flow velocity and hydraulic loading intensify rotor–stator interaction and may amplify local jet–wake effects and flow nonuniformity, resulting in stronger modulation of the excitation force. As a result, the vibration response changes from relatively steady periodic motion to a more complicated state characterized by impulsive components, waveform distortion, and beat-like modulation. In addition, the multistage structure of the pump further strengthens this nonlinear behavior. Because the hydraulic disturbance generated in one stage can propagate through the shaft system and casing structure to adjacent stages, the vibration response is determined not only by a single local excitation source but also by the cumulative superposition of multistage flow-induced forces and structural transmission effects. Therefore, once the pump operates away from the design condition, the coupled action of flow instability, rotor–stator interaction, and structural dynamic sensitivity can significantly increase waveform irregularity and fault risk. Although the present study focuses on pump-level vibration behavior, persistent high-frequency pressure pulsations may be transmitted through the fluid column and connected structural paths to the subsea wellhead system and the casing-cement-formation assembly. Such sustained dynamic disturbance may increase the long-term risk of fatigue damage and deterioration of cement-sheath sealing integrity, which is critical to the service life and safe operation of oil and gas wells^50^.

It should also be noted that cavitation was not directly reproduced or identified in the present experimental and numerical setup. Therefore, the nonlinear vibration characteristics reported in this study are mainly interpreted as the result of flow instability, unsteady hydraulic excitation, and their coupling with mechanical faults, such as looseness or misalignment, rather than being directly attributed to cavitation. All three directions (X, Y, Z) are dominated by intermittent impact pulses, rather than steady harmonic vibrations. This typical “ringing” pulse​ is a typical time-domain signature of mechanical looseness, strongly indicating potential fastener loosening issues in the pump or its base structure. However, actual drilling fluids are generally non-Newtonian, and their viscosity, yield stress, and rheological damping may modify the amplitude and high-frequency content of the observed “ringing” response. Therefore, these results should be interpreted as a baseline evaluation under simplified water-test conditions, and the quantitative effects of drilling fluid rheology require further study. As the flow rate gets close to the rated condition, the vibration performance improves significantly, entering a relatively optimal operating range. In practical operation, it is recommended to stabilize the pump within this flow range to ensure smoother operation and minimize fault risk. Furthermore, prolonged operation under extremely low or high flow conditions should be avoided to prevent mechanical faults caused by flow oscillations​ and significant impact loads​.

### Fault identification and risk assessment

**Fig. 13:**
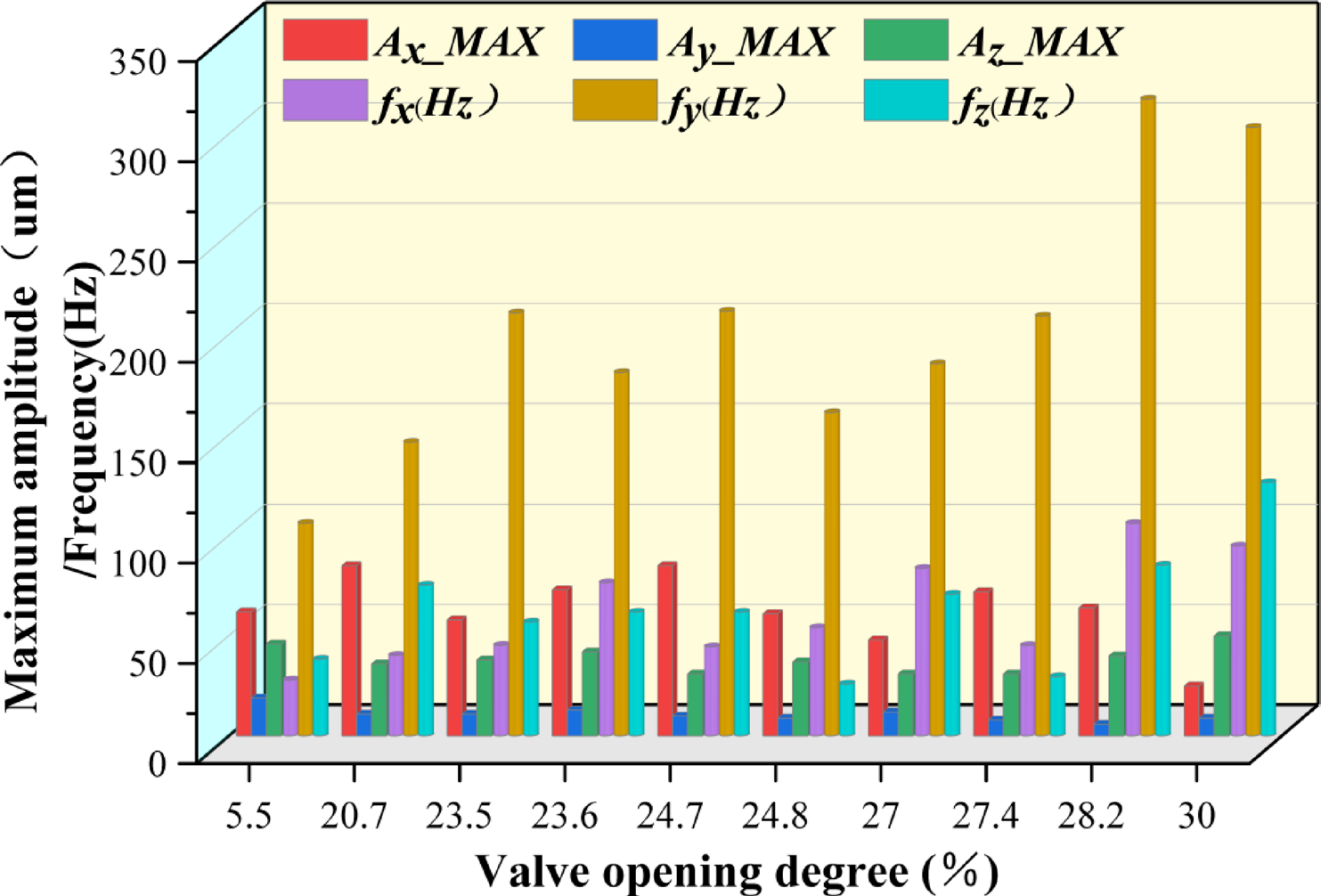
Maximum amplitudes and corresponding characteristic frequencies under different valve openings.

Under harsh deep-sea operating conditions, a centrifugal pump is susceptible to various fault types, including rotor unbalance, shaft misalignment, mechanical looseness, bearing and lubrication failures, rotor-stator friction, and shaft bending. To accurately identify potential fault risks under different operating conditions and establish a mapping relationship between vibration characteristics and potential fault modes, the maximum amplitudes (*A*_*x*_*_MAX*, *A*_*y*_*_MAX*, *A*_*z*_*_MAX*) and their corresponding characteristic frequencies (*f*_*x*_, *f*_*y*_, *f*_*z*_) along the X, Y, and Z axes were extracted under various flow conditions (corresponding to valve openings ranging from 5.5 to 30%). The results are illustrated in Fig. 13.

The six-stage centrifugal pump uses water-lubricated thrust bearings, which generally do not experience bearing faults. By comparing the vibration characteristic parameters (RMS, Cf, maximum amplitude, and characteristic frequency) with the vibration data at the rated flow rate, the potential faults and corresponding characteristics of the six-stage centrifugal pump under different flow conditions were identified, as summarized in Table 6.

**Table 6:** ​ Fault and characteristic classification.

No.	Fault type	RMS characteristics	Cf characteristics	Maximum amplitude characteristics	Characteristic frequency	Time-domain waveform characteristics
1	Unbalance	RMS_X, Z > > RMS_Y	Cf ≈ 3–3.5	Ax_MAX stable	f = 1×fr	Regular sine wave
2	Misalignment	RMS_Y increases significantly and ≈ RMS_X, Z	Cf ≤ 4	Ay_MAX↑ ≈ Ax_MAX	f = 2×fr (dominant)	Waveform distortion
3	Mechanical Looseness	RMS unstable in all directions	Cf normal or slightly high	A_MAX fluctuates significantly	f = n×fr (*n* ≥ 3)	Jumping/Ringing
4	Rubbing	High-frequency energy	Cf increases	Limiting effect	f = (1/2)×fr + wide frequency	Clipped/Flat-topped

To systematically evaluate the mechanical condition of the six-stage centrifugal pump under different flow conditions, this study comprehensively identified and assessed the risks and fault modes across ten flow conditions. By linking the joint indicator behavior with time-domain waveform characteristics and operating conditions, a fault-risk mapping framework for the pump across the full flow range was established. The assessment was based on measured data of RMS, Cf, maximum amplitude, and characteristic frequency, combined with time-domain waveform characteristics and the fault classification criteria outlined in Table 6. Appendix A summarizes the detailed evaluation results for each condition, including the flow rate at each valve opening, vibration severity levels along the X, Y, and Z axes, Cf risk levels, overall risk assessment, and fault type identification. The overall risk assessment divides vibration severity and Cf risk levels into three grades: A (low risk), B (medium risk), and C (high risk). Grade A indicates that all indicators are within acceptable limits; Grade B signifies that at least one direction reaches a level requiring attention but without immediate fault; and Grade C indicates the presence of significant fault characteristics (e.g., Cf ≥ 4.0 or vibration deterioration in multiple directions).

Analysis of the data in Appendix A reveals clear trends in the vibration state and dominant fault modes of the pump unit as the flow rate changes. Under low-flow conditions, vibration already presents a high risk, primarily characterized by severe vibration in the X-direction (radial horizontal). This is typically caused by intense flow instability at low flow rates, which can induce or worsen mechanical looseness and misalignment issues. As the flow rate approaches the rated condition (Q = 126 m³/h), the fluid energy supply stabilizes, and the flow pattern improves. This results in smoother vibration waveforms with enhanced periodicity, maintaining the risk at a medium level. Within this optimal operating range, rotor unbalance becomes the dominant fault factor. When the flow rate further increases into the high-flow region, the vibration characteristics deteriorate again, and the risk level rises. At this stage, the excessive flow rate significantly increases the radial force on the impeller, potentially worsening shaft misalignment and reactivating significant mechanical looseness characteristics, accompanied by increased waveform disorder. This is particularly significant because at Q = 260 m³/h, the Crest Factor (Cf) exceeds the risk threshold of 4.0. This is a clear indicator of rotor-stator friction, signaling a high operational risk under this condition that could lead to rapid equipment damage.

It should be noted that the fault-type interpretation in this study is inferential in nature. The identification of fault modes was not based on controlled seeded-fault experiments or independently verified fault cases but on the consistency between the observed vibration signatures and the diagnostic characteristics commonly reported in previous studies. Therefore, the corresponding fault classifications should be understood as qualitative fault-risk indications under different operating conditions. The proposed method is primarily developed for vibration signals acquired under controlled test conditions, and its reliability may decrease under low-SNR conditions, as background noise can distort both the RMS of vibration velocity and the Crest Factor (Cf) of acceleration, especially for weak impulsive characteristics. Therefore, additional denoising, sensor optimization, or robust characteristic extraction would be required prior to direct application in deep-sea environments with strong acoustic interference.

## Conclusions

This study systematically investigated the vibration evolution and fault risks of a six-stage centrifugal lift pump. A Fluid-Structure Interaction (FSI) dynamics model was first established to analyze the distribution of fluid excitation forces inside the pump and the structural modal response up to the sixth order. A vibration test platform was then constructed to conduct performance and vibration tests across the full flow range (0–260 m³/h, corresponding to valve openings of 5.5–30%) at the rated speed of 1450 rpm. Vibration signals at the pump outlet flange were collected under various conditions to analyze time-domain characteristics and evolution patterns, assess vibration risks under off-design flow conditions, and establish a fault identification method. The main conclusions are summarized as follows:

1. Under rated conditions, the flow field inside the pump remains stable, although local vortices at the impeller-diffuser junction induce high frequency pressure pulsations. Modal analysis confirms sufficient safety margins between the first six natural frequencies of the pump (63.96–195.74 Hz) and the excitation frequencies at rated operation (shaft frequency at 24.17 Hz, blade-passing frequency at 72.5 Hz). However, the pump passes through the first (1278.4 rpm) and second (1295.2 rpm) critical speed regions during startup, which should be traversed rapidly to avoid resonance.
2. The high correlation between simulated and experimental head-flow (H-Q) curves across the full flow range validates the reliability of the CFD model. The vibration parameters, including amplitude (≤ 38 μm), velocity RMS (< 3.9 mm/s), and peak factor Cf (< 3.8), all remain within safe thresholds under rated conditions (126 m³/h). In contrast, particularly at low (Q ≤ 28 m³/h) and high (Q ≥ 237 m³/h) flow rates, off-design conditions exhibit strongly nonlinear vibration deterioration, with significant increases in RMS and Cf, indicating higher risks.
3. Vibration risk and fault modes evolve with flow rate. At a low flow rate, “ringing” waveforms and periodic impulses indicate misalignment and mechanical looseness. Near the design point (130–156 m³/h), vibration stabilizes, with the dominant risk shifting to acceptable rotor unbalance. At a high flow rate, waveform distortion and “beat vibration” indicate potential misalignment, looseness, or rotor-stator contact. Accordingly, the optimal operating range is identified as a valve opening of 23.7–24.8%, corresponding to a flow rate of 130–156 m³/h, where vibration is minimal and risk is controllable.

This study has certain limitations in fault interpretation. The associations between waveform characteristics and specific fault types were not validated through controlled fault simulation tests or independent diagnostic methods, such as alignment inspection, disassembly examination, or comparison with labeled fault cases. Therefore, the proposed framework is more suitable for vibration risk screening and preliminary fault trend assessment than for definitive fault diagnosis. Future work should focus on validating these interpretations through known fault experiments and independent diagnostic measurements.

## Data Availability

All data are available from the corresponding author upon reasonable request.

## Appendix A

**Table:**

Valve opening	Flow rate Q(m³/h)	Vibration severity level (RMS-Based)	Cf risk level	Overall risk assessment	Fault identification
​5.5%​​	0	X: C Y: C Z: B	X: A Y: A Z: B	​C	2,3
20.7%​​	28	X: C Y: C Z: B	X: B Y: A Z: B	C	2,3
​23.5%​​	65	X: C Y: B Z: B	X: A Y: A Z: B	​B	1,3
​23.6%​​	80	X: C Y: B Z: B	X: B Y: A Z: B	​B	1,3
​24.7%​​	130	X: C Y: B Z: B	X: B Y: A Z: A	​B	1
​24.8%​​	156	X: C Y: B Z: B	X: A Y: A Z: A	​B	1
​27%​​	178.8	X: C Y: B Z: B	X: B Y: A Z: B	​B	1,2
​27.4%​​	193	X: C Y: B Z: B	X: B Y: A Z: B	​B	2,3
​28.2%​​	237	X: C Y: C Z: B	X: A Y: A Z: B	C	2,3
​30%​​	260	X: C Y: C Z: B	X: C Y: A Z: A	​C	2,4
